# Suppressor effects of carrots on azoxymethane/dextran sulfate sodium-induced colon cancer according to cultivation method

**DOI:** 10.3389/fimmu.2025.1554801

**Published:** 2025-04-11

**Authors:** Yanni Pan, Yeon-Jun Lee, Jin Hyeop Kim, Min Ji Song, KyuBum Kwack, Seung-Hwan Park, Sin-Il Sin, Ji Hyung Chung, Kun-Young Park

**Affiliations:** ^1^ Collaborative Innovation Center for Child Nutrition and Health Development, Chongqing Engineering Research Center of Functional Food, Chongqing Engineering Laboratory for Research and Development of Functional Food, Chongqing University of Education, Chongqing, China; ^2^ Department of Biotechnology, CHA University, Seongnam, Republic of Korea; ^3^ Department of Biomedical Science, CHA University, Seongnam, Republic of Korea; ^4^ Organic Anti-Cancer Agriculture Institute, iCOOP Natural Dream Company, Goesan-gun, Chungcheongbuk-do, Republic of Korea; ^5^ Graduate School of Integrative Medicine, CHA University, Seongnam, Republic of Korea

**Keywords:** carrots, deep sea water minerals (DSWM), colorectal cancer (CRC), inflammation, apoptosis, gut microbiome

## Abstract

**Introduction:**

This study investigated the suppressor effects of carrots depending on cultivation method on AOM/DSS-induced colon cancer in mice by examining cell apoptosis, inflammation response, and metabolites. Carrots grown using different fertilizers significantly suppressed tumor development by modulating cell apoptosis and inflammatory responses in our experimental settings.

**Methods and Results:**

Naturaldream Fertilizer Carrot (NFC) cultivated with deep sea water minerals (DSWM) showed effectively increased the expression of apoptosis-related genes and proteins including p53, p21, Bim, Bad, Bax, Bak, Caspase 9, and Caspase 3 in colon tissue, while inhibiting the production of inflammatory factors and related genes and proteins such as TNF-a, IL-1b, IL-6, IFN-g, NF-kB, and iNOS in serum, spleen cells, and liver tissues. Intestinal microbiota analysis revealed a distinct composition in mice receiving carrots compared to the control group, with accumulation of intestinal microorganisms such as *Lachnospiraceae,* and *Mucispirillum schaedleri* closely associated with anti-tumor effects.

**Discussion and Conclusion:**

Overall, our results indicate that carrots, especially carrots grown with DSWM fertilizers, play a crucial role in inhibiting AOM/DSS-induced colon cancer in mice by regulating cell apoptosis and inflammation responses. The present findings provide valuable insights for further exploration of carrots depending on the cultivation method, as a potential dietary source against colon cancer.

## 1 Introduction

Colorectal cancer (colon or rectal cancer, CRC) is a common malignant tumor originating from epithelial cells in the colon or rectum (1). And also, CRC is one of the most common cancers worldwide, it has high incidence and mortality rates in many regions (2). The pathogenesis of CRC is complex, involving multiple factors such as genetics, environment, and lifestyle (3). Unhealthy habits like high-fat and/or high-sugar diets, smoking, and alcohol consumption are associated with an increased risk of CRC (4). In early stages, CRC usually presents no obvious symptoms. As the tumor grows, patients may experience symptoms such as rectal bleeding, abdominal pain, and weight loss, which can worsen in late stages, leading to serious complications like intestinal obstruction, significantly impacting patients’ quality of life and survival (5). Compared to costly and side-effect-prone drugs, food-based targeted therapies can prevent and manage diseases by adjusting diet, utilizing bioactive compounds in food to modulate physiological processes and having potential for the prevention and management of various chronic diseases (6, 7). In addition, a high-fiber diet can prevent constipation and CRC, and the sulfides in vegetables may have inhibitory effects on cancer (8). The Azoxymethane/Dextran Sodium Sulfate (AOM/DSS) induced CRC mouse model is commonly used for researching the underlying mechanisms, preventive strategies, and treatment of CRC (9). AOM is a mutagen that can induce DNA damage, while DSS can induce intestinal inflammation and damage, leading to the breakdown of the intestinal mucosal barrier (10, 11). Their combination can simulate the development of CRC. Hence, this study will utilize the AOM/DSS-induced model to simulate the development of CRC, explore potential therapeutic targets for carrots depending on fertilizer, and develop preventive and treatment strategies.

Carrot (*Daucus carota*) is a vegetable rich in dietary fiber, vitamins, minerals, and antioxidants, having potential as food-based targeted therapy, given its various health benefits, including its potential anticancer effects (12, 13). One of the most unique components in carrots is beta-carotene, thought to have significant anticancer properties as it can neutralize free radicals and reduce oxidative stress, protecting cells from oxidative damage and reducing the likelihood of mutations (14, 15). Moreover, beta-carotene can regulate apoptosis (programmed cell death), something crucial for inhibiting the growth and spread of cancer cells (16). Additionally, beta-carotene can enhance the immune function, promote the activity of immune cells, aiding in identifying and destroying cancer cells, and also believed to slow tumor growth by inhibiting formation of new blood vessels, restricting blood supply to the tumor (17–19). This suggests that carrots can significantly suppress the occurrence of colon cancer, and this study seeks to determine whether these functions of carrots can be improved depending on the cultivation method.

The content of minerals and phytochemicals in plants is influenced by various factors, including cultivation methods, fertilizers, and the environment (20). Currently, the emphasis on healthy eating has increased the attention on organic foods free of chemical pesticides (21). Recently, due to soil contamination caused by the continuous use of chemical fertilizers, various methods of growing crops without using them are being developed. Organic cultivation emphasizes the ecosystem health, utilizing natural organic fertilizers and pesticides such as animal and plant manure and natural insect repellents, reducing reliance on artificial chemicals, promoting soil health and ecological balance to provide healthier and environmentally friendly agricultural products (22, 23). In addition, for organic cultivation and to supplement insufficient nutrients, deep sea water minerals containing many types of minerals such as Mg, Ca, Cl, Na, K, Se, and V are used in agriculture (24). Deep sea water minerals are used in agriculture in the United States, Japan, Taiwan, and South Korea are continuously developing the technology and practical application of resources (25). For instance, the Carbon Healing Agricultural Research Institute (South Korea) has developed cultivation methods that incorporate natural minerals based on organic farming (26, 27). To investigate whether this newly developed cultivation method can enhance crop bioactivity, we compared the antioxidant, anticancer, and anti-inflammatory abilities of carrots grown through conventional, sea water, trace element, and naturaldream methods.

The complex interaction between the gut microbiota and CRC is well recognized (28). First, the gut microbiota plays a crucial role in maintaining intestinal immune balance and mucosal barrier function. Its imbalance may lead to intestinal mucosal inflammation and damage, consequently promoting the development of CRC (29, 30). Secondly, some studies suggest that potentially carcinogenic bacteria or bacterium-related metabolites (such as cholesterol metabolites, lipopolysaccharides, etc.) may induce intestinal inflammation, thereby increasing the risk of CRC (31). Additionally, the gut microbiota can influence the host’s metabolism and nutritional status, thereby affecting the development of CRC (32). There is a close and intricate relationship between CRC and gut microbiota, where the balance of gut microbiota plays a crucial role in the development of CRC. Therefore, adjusting the composition of gut microbiota to maintain its balance may help in the prevention and treatment of CRC. Future studies, understanding the intricate relationship between CRC and the gut microbiota following carrot consumption will be crucial for developing innovative and more effective strategies for the prevention and treatment of colorectal cancer.

## 2 Materials and methods

### 2.1 Sample preparation

The carrots used in this experiment were cultivated at the Carbon Healing Agriculture Research Institute in Gurye-gun, Goesan, Chungcheongbuk-do, Republic of Korea, under iCOOP Natural Dream Company. Nutrient management standards were created based on the crop cultivation manual provided by the Rural Development Administration. In order to supply the minimum nutrients required for crops, the input amounts of basic fertilizer, top fertilizer, nitrogen (N), phosphorus (P), calcium (Ca), and boron (B) were the same. Carrots were classified into four categories according to the type of fertilizer used. These include conventional fertilizer carrot (CFC) using pure water; Seawater fertilizer carrot (SFC) grown using seawater collected from the waters off Asan Bay, South Korea; trace element fertilizer carrot (TFC); and Naturaldream Fertilizer carrot (NFC) grown using deep sea water minerals (DSWM). Naturaldream fertilizer is a fertilizer for crop growth and is registered as an organic farming material by the National Agricultural Products Quality Management Service of Korea. The mineral components of each fertilizer were analyzed at the Korea Quality Testing and Research Institute (Suwon, Gyeonggi-do, South Korea). The Seawater Fertilizer contained 6,879 mg/kg of Sodium (Na), 808 mg/kg of Magnesium (Mg), 282 mg/kg of Calcium (Ca), 261 mg/kg of Potassium (K), 1.11 mg/kg of Iron (Fe), and 1,700 mg/kg of Chlorine (Cl). The Trace Element Fertilizer was found to have 1,339 mg/kg of Na, 3,432 mg/kg of Mg, 698 mg/kg of Ca, 9,784 mg/kg of K, 23,938 mg/kg of Fe, 7.37 mg/kg of Phosphate (P(PO_4_)), and 8,182 mg/kg of Silicon (Si). Lastly, the Naturaldream Fertilizer contained 9,565 mg/kg of Na, 60,000 mg/kg of Mg, 49.5 mg/kg of Ca, 11,588 mg/kg of K, 10.8 mg/kg of P(PO_4_), 0.61 mg/kg of Si, 14,700 mg/kg of Cl, 27 mg/kg of N, and 179 mg/kg of B (27).

### 2.2 Production of samples for oral administration to mice

The harvested carrot samples were freeze-dried at -80°C using an freeze dryer (FDS-7012, Operon, Korea). The freeze-dried carrot powder was then extracted with ethanol, using 10 times the weight of the freeze-dried carrot powder (mass/weight ratio) at room temperature for 24 hours. After filtration with a filter paper, the residue was subjected to repeated extraction for 24 h. The filtrates from the two extractions were then combined and concentrated using a vacuum concentrator to obtain a nontoxic carrot extract with a yield of 6.1%. According to a cohort study conducted by Deding et al. (2020)., it was confirmed that when the average daily carrot intake exceeded 32 g, the occurrence of CRC decreased by about 17% (33). In order to relate this to animal testing, it is assumed that a person weighing 60 kg per day consumes 32 g/day, and the conversion coefficient for body surface area of ​​humans and mice was calculated as 12.3 times. The daily intake of a mouse of 25 g per day is calculated to be 164 mg/day, and when calculated using the 6.1% yield from actual ethanol extraction, it is calculated to be 10.00 mg/day. Since the daily oral dose for mice is 0.2 mL, the extracts were dissolved in physiological saline to a concentration of 50 mg/mL and subsequent experiments were conducted. This extract was dissolved to a concentration of 50 mg/mL using physiological saline for subsequent experiments.

### 2.3 Animal experiment design

**Figure 1:**
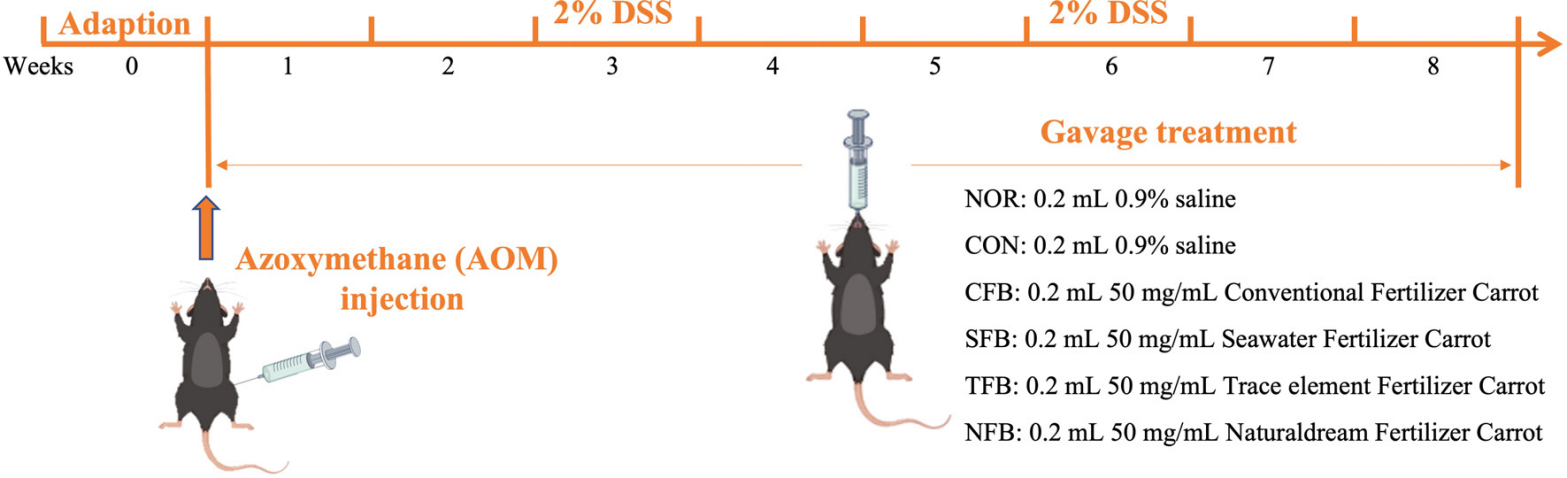
Timeline of the experiment of this study.

The animals used in this experiment were 6-week-old male C57BL/6 mice (20 g) purchased from Orientbio, South Korea. The mice were housed in SPF rooms with a controlled 12-h light/dark cycle at a temperature of 23°C ± 2°C and humidity of 50% ± 5%. During the 1-week acclimatization period, mice were provided with water and feed ad libitum. Then, mice were randomly divided into 6 groups (n = 10 per group) for the experiment, which lasted 8 weeks. Gavage was performed daily at 0.2 mL/20 g body weight (bw). Both normal (NOR) and control (CON) groups were administered with saline, while the Conventional Fertilizer Carrot (CFC), Seawater Fertilizer Carrot (SFC), Trace element Fertilizer Carrot (TFC), and Naturaldream Fertilizer Carrot (NFC) groups received 50 mg/mL of their respective extracts. Except for the NOR group, on the first day of the experiment, the remaining mice were intraperitoneally injected with 10 mg/kg b.w. Azoxymethane (AOM, Sigma, St. Louis, MO, USA). In addition, they got a 2% dextran sulfate sodium (DSS, MPBio, Solon, OH, USA) solution in their drinking water on the third and sixth weeks (**Figure 1**) (27). Throughout the experiment, the bw, activity levels, and stool conditions were monitored daily to confirm CRC induction. At the end of the experiment, the mice were euthanized with CO_2_ gas, and blood, liver, spleen, and colon tissues were collected for further analysis. This animal experiment design was approved by the CHA University Animal Ethics Committee (Approval No. IACUC-220126).

### 2.4 Detection of total phenol, and total flavonoid content in carrot samples

Total phenol (TP) content: Phosphomolybdic acid and phosphotungstic acid are easily reduced by phenolic compounds and turn blue under alkaline conditions; thus, we used a folin-ciocalteu reagent to detect TP content. The standard curve was drawn using Gallic acid as standard (standard concentration, 0.03125–1 mg/mL), and the TP content of samples was calculated accordingly.

Total flavonoid (TF) content: According to the principle of color change in the reaction of NaOH and flavonoids, we used quercetin as the standard (standard concentration, 0–1,280 μg/mL) to draw the standard curve, and the TF content of samples was calculated accordingly.

### 2.5 Histopathological observation of mouse colon and spleen tissues

At the end of the 9-week experimental period, the mice were sacrificed using CO_2_ gas, and their organs were harvested. Approximately 0.5 cm length of colon tissue and a 0.5 cm² piece of spleen tissue were collected from the mice and immersed in a tissue fixative. The tissues were then fixed, dehydrated, embedded in paraffin, sectioned, and stained with H&E. Morphological structures were observed using a light microscope.

### 2.6 Detection of cytokine levels in mouse serum and splenocyte culture supernatant

To collect the serum, blood was centrifuged at 13,000 rpm for 15 min at 4°C and stored at −80°C for future use. The spleen was minced and filtered, and red blood cells were removed using an RBC lysis buffer. The resulting suspension was then seeded in a 6-well plate at a concentration of 1 × 10^6^ cells/mL and incubated for 24 h to form a supernatant. For cytokine measurement, TNF-α, IL-1β, IL-6, IFN-γ, and IL-10 concentrations were measured using ELISA kits (BioLegend, San Diego, California, USA) according to manufacturer’s instructions.

### 2.7 RT-qPCR

mRNA expression in the colon and liver was quantified using a SYBR green-based RT-qPCR. Approximately 100 mg of colon and liver tissues were homogenized, and total RNA was extracted using Trizol. RNA concentration was determined using a microspectrophotometer. Reverse transcription was performed using a reverse transcriptase first strand cDNA synthesis kit to obtain cDNA templates. Subsequently, 10 μL of SYBR Green PCR Master Mix (Applied Biosystems, USA) was added. The target was amplified in the StepOnePlus real-time PCR system using 1 μL of forward primer, 1 μL of reverse primer, 1 μL of cDNA template, and 7 μL of nuclease-free water. The amplification conditions were as follows: 95°C for 3 min for initial denaturation; 40 denaturation cycles at 95°C for 15 s, annealing at 60°C for 30 s, extension at 72°C for 15 s; and a final melt curve step at 95°C for 30 s, 60°C for 30 s, and 95°C for 15 s. The relative expression levels of each gene were calculated using the 2^−ΔΔCT^ method, where CT represents the cycle threshold and using β-actin as internal reference gene. **Tables 1**, **2** lists the primer sequences used in this study.

**Table 1:** Primer sequences of inflammation-related genes.

Gene Name	Primer Sequence
NF-κB p50	F: 5’-CACCTAGCTGCCAAAGAAGG-3’
	R: 5’-GCAGGCTATTGCTCATCACA-3’
NF-κB p65	F: 5’-ATGGCAGACGATGATCCCTAC-3’
	R: 5’-CGGAATCGAAATCCCCTCTGTT-3’
IκB	F: 5’-AGGACGAGGAGTACGAGCAA-3’
	R: 5’-GTCTCCCTTCACCTGACCAA-3’
iNOS	F: 5′-ATGGCTTGCCCCTGGAA-3′
	R: 5′-TATTGTTGGGCTGAGAA-3′
IL-6	F: 5′-ATGAAGTTCCTCTCTGCAA-3′
	R: 5′-AGTGGTATCCTCTGTGAAG-3′
IFN-γ	F: 5’-GCTTTGCAGCTCTTCCTCAT-3’
	R: 5’-GTCACCATCCTTTTGCCAGT-3’
IL-10	F: 5’-CCAAGCCTTATCGGAAATGA-3’
	R: 5’-TTTTCACAGGGGAGAAATCG-3’
GAPDH	F: 5’-AGGTCGGTGTGAACGGATTTG-3’
	R: 5’-GGGGTCGTTGATGGCAACA-3’

**Table 2:** Primer sequences of apoptosis-related genes.

Gene Name	Primer Sequence
p53	F: 5’-ATGGAGGAGCCGCAGTCAGA-3’
	R: 5’-TGCAGGGGCCGCCGGTGTAG-3’
p21	F: 5’-ATGTCAGAACCGGCTGGGG-3’
	R: 5’-GCCGGGGCCCCGTGGGA-3’
Bim	F: 5’-AGATCCCCGCTTTTCATCTT-3’
	R: 5’-TCTTGGGCGATCCATATCTC-3’
Bad	F: 5’-CAATGACCCCTTCATTGACC-3’
	R: 5’-GACAAGCTTCCCGTTCTCAG-3’
Bak	F: 5’-TCTGGCCCTACACGTCTACC-3’
	R: 5’-AGTGATGCAGCATGAAGTCG-3’
Bax	F: 5’-TGCTTCAGGGTTTCATCCAG-3’
	R: 5’-GGCGGCAATCATCCTCTG-3’
Bcl-2	F: 5’-AAGATTGATGGGATCGTTGC-3’
	R: 5’-GCGGAACACTTGATTCTGGT-3’
Bcl-xL	F: 5’-GCTGGGACACTTTTGTGGAT-3’
	R: 5’-TGTCTGGTCACTTCCGACTG-3’
Caspase-9	F: 5’-CTAGTTTGCCCACACCCAGT-3’
	R: 5’-CTGCTCAAAGATGTCGTCCA-3’
Caspase-3	F: 5’-TTTTTCAGAGGGGATCGTTG-3’
	R: 5’-CGGCCTCCACTGGTATTTTA-3’
GAPDH	F: 5’-AGGTCGGTGTGAACGGATTTG-3’
	R: 5’-GGGGTCGTTGATGGCAACA-3’

### 2.8 Western Blot

To homogenize 100 mg of colon and liver tissue samples, we added 1 mL of radioimmunoprecipitation assay (RIPA) buffer (Invitrogen, Carlsbad, CA, USA), followed by centrifugation at 4°C and 12,000 rpm for 10 min. Then, the supernatant was recovered to quantify the protein concentration using the Bradford assay. The samples were then concentrated to 80 μg/mL, mixed with sample buffer at a ratio of 3:1, and heated at 100°C for 10 min. Then, the samples were premixed with the prestained protein ladder on a vertical 12% Mini-PROTEAN^®^ TGX™ Precast Protein Gel (BIO-RAD, Hercules, CA, USA) for electrophoresis. Next, proteins were transferred onto a polyvinylidene difluoride (PVDF) membrane using the iBlot™ transfer stack and iBlot™ 2 gel transfer device (Invitrogen, Carlsbad, CA, USA) at 25 V for 5 min. Subsequently, the PVDF membrane was blocked in PBS-T buffer containing 0.1% Tween-20 and 5% skim milk for 1 h, followed by washing three times with PBS-T and once with PBS. Then, samples were incubated with primary antibodies overnight at 4°C. The primary antibodies against p21, Bax, Bcl-2, Caspase 3, IκB-α, IL-6, and β-actin were obtained from Santa Cruz (Dallas, Texas, USA). After washing, samples were incubated with secondary antibodies for 2 h at room temperature. Finally, the PVDF membrane was immersed in Clarity TM Western ECL substrate (BIO-RAD, Hercules, CA, USA) for 30 s, followed by imaging using the Amersham Imager 680 system (GE Healthcare Life Sciences, Chicago, IL, USA).

### 2.9 Measurement of natural killer (NK) cell activity

Spleen cells were dispersed in a 96-well plate at a concentration of 1 × 10^6^ cells/well, as effector cells. YAC-1 cells were used as target cells, with an effector-to-target cell ratio of 5:1 in each well. The YAC-1 cells utilized in this study were obtained from the American Type Culture Collection (Manassas, Virginia, USA) and cultured in RPMI 1,640 medium supplemented with 10% fetal bovine serum (FBS) and 1% penicillin-streptomycin (PS), and subcultured 3–4 times per week for future experiments. Samples were treated at specific concentrations. Cell cytotoxicity against YAC-1 cells was assessed using the EZ-LDH assay kit (DoGenBio, Geumcheon-gu, Seoul, South Korea) according to manufacturer’s instructions. LDH levels were measured to evaluate the cytotoxicity of the samples against YAC-1 cells.

### 2.10 Gut microbiota analysis

Genomic DNA was extracted from mouse fecal samples using the magnetic bead method and the Soil and Fecal Genomic DNA Extraction Kit (TIANGEN BIOTECH CO., LTD. Beijing, China). Subsequently, 1% agarose gel electrophoresis was performed to assess DNA purity and concentration. An appropriate amount of sample DNA was placed in a centrifuge tube and diluted with sterile water to 1 ng/μL. All PCR mixtures were prepared by adding 15 μL of Phusion™ High-Fidelity PCR Master Mix (New England Biolabs), 0.2 μM primers (16S V4 region primers 515F and 806R), and 10 ng of the genomic DNA template. The PCR amplification protocol included an initial denaturation at 98°C for 1 min, followed by 30 cycles of denaturation at 98°C for 10 s, annealing at 50°C for 30 s, and extension at 72°C for 30 s, with a final extension phase at 72°C for 5 min. PCR products were analyzed by electrophoresis on a 2% agarose gel. Qualified PCR products underwent magnetic bead purification, were quantified using enzymatic methods and pooled at equimolar concentrations based on the PCR product concentration. After thorough mixing, the PCR products were subjected to another round of electrophoresis on a 2% agarose gel and target bands were purified using a universal DNA purification kit (Tiangen Biotech Co. Ltd., Beijing, China). Library construction was carried out using the NEBNext^®^ Ultra™ II FS DNA PCR-free Library Prep Kit (New England Biolabs, Ipswich, Massachusetts, USA). The constructed libraries were quantified with Qubit and Q-PCR, and after passing a quality control, PE 250 sequencing was performed on the NovaSeq 6,000 platform. Each sample data were extracted from the raw data based on the Barcode sequence and PCR amplification primer sequence. After trimming the Barcode and primer sequences, the reads of each sample were assembled using FLASH (Version 1.2.11, http://ccb.jhu.edu/software/FLASH/), resulting in assembled sequences as the Tags, which were subjected to rigorous filtering using fastp software (Version 0.23.1) to obtain high-quality Clean Tags. After processing, the Tags obtained underwent removal of chimeric sequences. Tags sequences were aligned with species annotation databases (Silva database https://www.arb-silva.de/for16S/18S, Unite database https://unite.ut.ee/forITS) to detect chimeric sequences and remove them to obtain Effective Tags (34).

### 2.11 Statistical analysis

Total phenols and flavonoids were analyzed using Graph Pad Prism 9.4.1 (GraphPad, SanDiego, CA, USA); the experimental data are expressed as mean ± standard deviation. Two-way ANOVA was used to compare data between groups (*p < 0.05, **p < 0.01, ***p < 0.001, ****p < 0.0001). The serum and tissue indices of each mouse were determined by conducting ≥3 parallel experiments, followed by calculating the mean values. Data analysis was performed using SPSS 22 software. Experimental results were expressed as mean ± standard deviation. Duncan’s multiple range test was employed to assess differences in mean values among groups using one-way analysis of variance. A significance level of *p* < 0.05 was considered significant.

## 3 Results

### 3.1 TP and TF content in carrot samples

**Figure 2:**
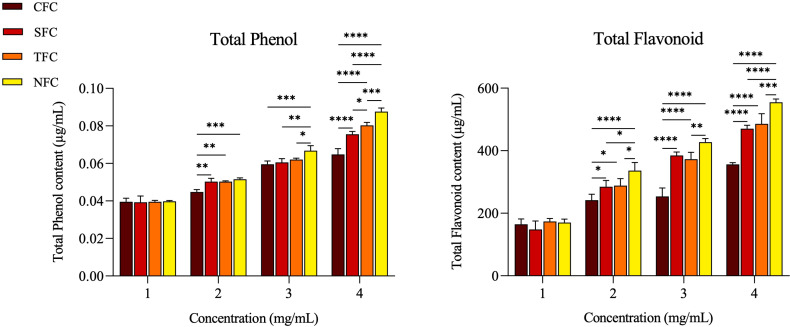
Total phenol, and total flavonoid contents by concentration of carrot. The *, **, ***, **** symbol means significantly different (*p* < 0.05), (*p* < 0.01), (*p* < 0.001), (*p* < 0.0001), respectively, by Two-way ANOVA.

The antioxidant effect of carrots is shown in **Figure 2**. The total phenolic (TP) and total flavonoid (TF) content of the four types of carrots (CFC, SFC, TFC, NFC) increased with higher carrot concentrations. Additionally, the TP and TF content in NFC were higher than in other carrots at 2 - 4 mg/mL concentrations (*p* < 0.05). These results indicate that Naturaldream carrots have superior antioxidant capacity to other carrots.

### 3.2 Impact of carrots on mouse body and organ weight, colon length, weight/length ratio, and tumor count

**Figure 3:**
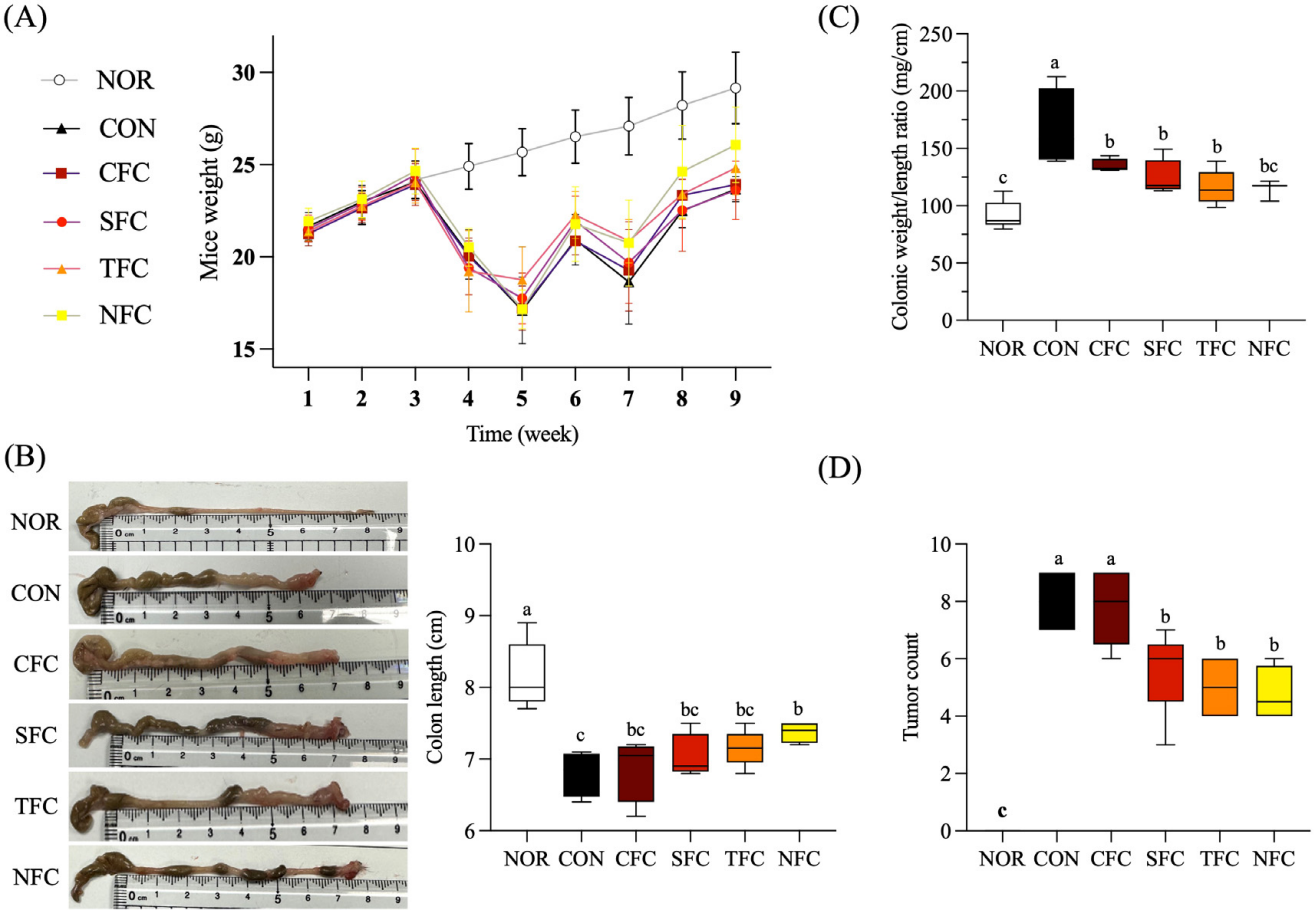
Effect of Carrot oral administration on body weight **(A)**, colon length **(B)**, colon weight/length ratio **(C)**, and tumor count **(D)** of C57BL/6 colon cancer mice induced by AOM/DSS. NOR: 0.9% saline solution; CON: AOM/DSS + 0.9% saline solution; CFC: AOM/DSS + Conventional Fertilizer Carrot extract 50mg/mL; SFC: AOM/DSS + Seawater Fertilizer Carrot extract 50 mg/mL; TFC: AOM/DSS + Trace element Fertilizer Carrot extract 50 mg/mL; NFC: AOM/DSS + Naturaldream Fertilizer Carrot extract 50mg/mL. Means with the different letters (a-c) above the bars are significantly different by Duncan’s multiple range test (*p* < 0.05).

Throughout the experiment, the body weight of mice in the NOR group steadily increased. In comparison, significant weight loss was observed in mice induced with AOM/DSS; an effect seemingly alleviated by consuming carrots. Particularly, the group of CRC mice fed with NFC had a body weight close to the normal group (**Figure 3A**). As shown in **Figure 3B**, compared to the NOR group, the colon length of mice in the CON group was significantly shorter after AOM/DSS treatment; in addition, the degree of colon shortening varied with the different carrot treatments. Among them, the colon length of mice in the CFC and SFC groups was similar, with NFC mice showing a colon length closest to the normal group, followed by TFC.

**Figure 3C** shows that, compared to the NOR group, AOM/DSS increased the colon weight/length ratio, while carrot intake reduced tumor incidence and maintained colon length, thereby lowering this ratio. Additionally, no tumors were found in the colon of NOR mice, while the CON group had the highest tumor occurrence (**Figure 3D**). Further, carrot treatment suppressed tumor occurrence to varying degrees, with NFC having the fewest tumors followed by TFC, despite the lack of significant differences between the two.

As shown in **Table 3**, the liver and spleen weight of mice in the CON group were significantly higher than in the NOR group, but lighter in mice consuming carrots and more similar to those in the NOR group. At the same time, there were no significant differences in kidney and testis weight among all groups, suggesting the lack of carrot toxicity on the kidneys and testis.

**Table 3:** C57BL/6 mice organ weight.

Weight (g)	NOR	CON	CFC	SFC	TFC	NFC
Liver	1.18 ± 0.12^ab^	1.25 ± 0.16^a^	1.26 ± 0.09^a^	1.07 ± 0.10^b^	1.13 ± 0.09^ab^	1.09 ± 0.06^b^
Spleen	0.07 ± 0.02^b^	0.16 ± 0.03^a^	0.13 ± 0.07^ab^	0.16 ± 0.07^a^	0.13 ± 0.08^ab^	0.09 ± 0.02^ab^
Kidneys	0.34 ± 0.03^a^	0.31 ± 0.01^ab^	0.29 ± 0.03^b^	0.28 ± 0.04^b^	0.30 ± 0.02^b^	0.30 ± 0.01^b^
Testis	0.18 ± 0.02^a^	0.15 ± 0.03^ab^	0.14 ± 0.02^b^	0.15 ± 0.03^ab^	0.14 ± 0.03^b^	0.14 ± 0.01^b^

Means with the different letters (a-b) next to the values are significantly different by Duncan’s multiple range test (*p* < 0.05).

### 3.3 Histopathological examination of mouse colon and spleen tissues

**Figure 4:**
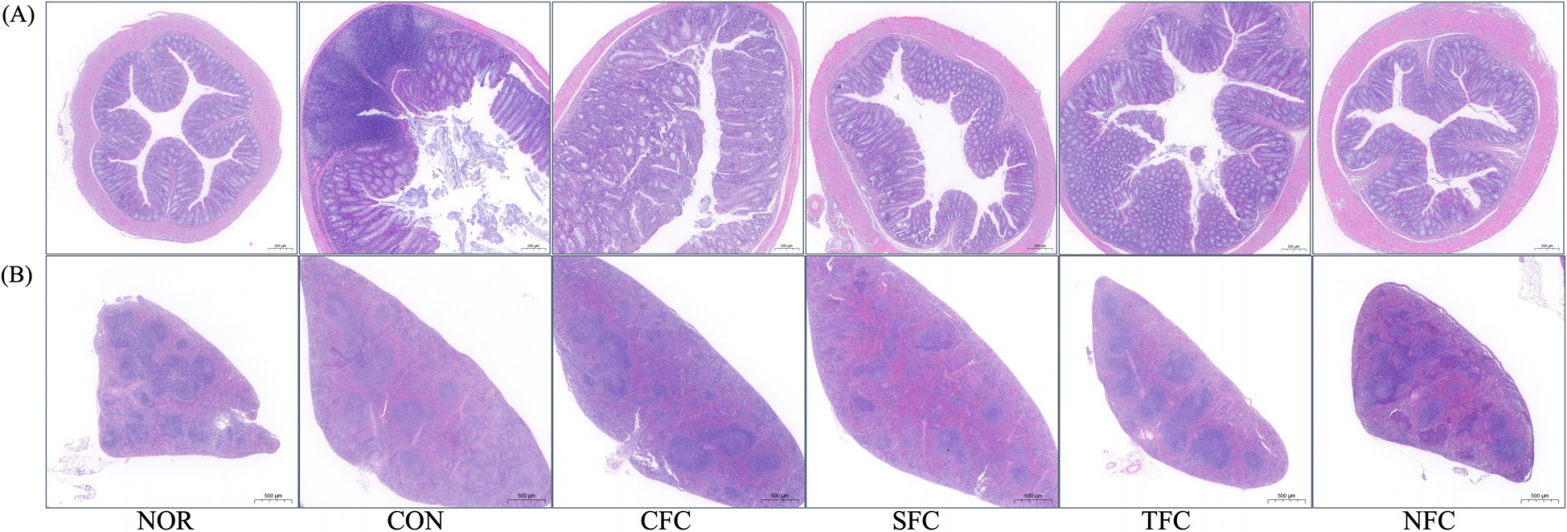
Morphological alterations in mice colon **(A)** and spleen **(B)** tissues following carrot sample administration. NOR: 0.9% saline solution; CON: AOM/DSS + 0.9% saline solution; CFC: AOM/DSS + Conventional Fertilizer Carrot extract 50mg/mL; SFC: AOM/DSS + Seawater Fertilizer Carrot extract 50 mg/mL; TFC: AOM/DSS + Trace element Fertilizer Carrot extract 50 mg/mL; NFC: AOM/DSS + Naturaldream Fertilizer Carrot extract 50mg/mL.

**Figure 4** shows the results of the histological examination of colon and spleen tissues stained with H&E in each experimental group. In the animal model for CRC inhibition, the direction of cancer or tumor development in colon tissues is generally observed to confirm the induction of colon cancer. In CON group, tumors developed in the mucosa in many parts of the colon. The CFC group exhibited some degree of cancer-related tissue orientation, while the SFC and TFC groups showed many areas of ulcer healing. Meanwhile, treatment with NFC significantly inhibited tumor development in AOM/DSS-induced CRC mice (**Figure 4A**). The spleen tissue from the normal group showed a normal spleen structure, including normal periarterial lymphatic sheaths, lymphatic follicles, and marginal sinuses. In the CON group, the overall spleen size increased, lymphatic follicles were reduced, and the lymphatic structures got distorted into diffuse white pulp. The groups fed with carrots exhibited a relatively normal spleen structure, with the NFC group showing the brightest white and red pulps, along with presence of macrophages as well as many follicles (**Figure 4B**).

### 3.4 Changes in inflammatory cytokine levels in mouse serum

**Figure 5:**
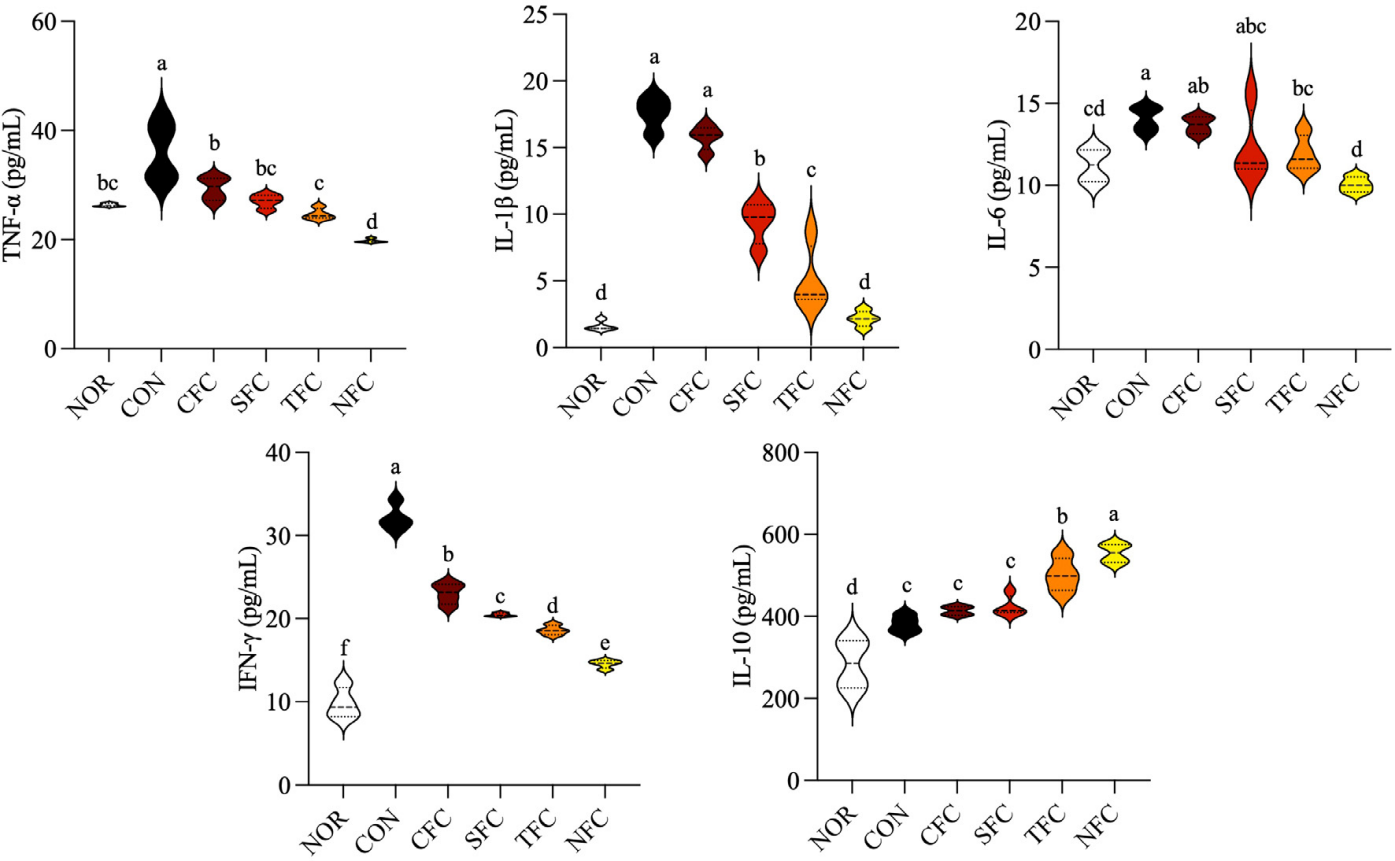
Levels of TNF-α, IL-1β, IL-6, IFN-γ, and IL-10 cytokines in mice serum. NOR: 0.9% saline solution; CON: AOM/DSS + 0.9% saline solution; CFC: AOM/DSS + Conventional Fertilizer Carrot extract 50mg/mL; SFC: AOM/DSS + Seawater Fertilizer Carrot extract 50 mg/mL; TFC: AOM/DSS + Trace element Fertilizer Carrot extract 50 mg/mL; NFC: AOM/DSS + Naturaldream Fertilizer Carrot extract 50mg/mL. Means with the different letters (a-f) above the bars are significantly different by Duncan’s multiple range test (*p* < 0.05).

The levels of inflammatory cytokines in mouse blood are shown in **Figure 5**. AOM/DSS induction significantly increased the levels of inflammatory cytokines TNF-α, IL-1β, IL-6, and IFN-γ in mouse serum. Conversely, carrot treatment decreased them, with the most effective inhibition observed in the NFC group, followed by the TFC group. Additionally, the serum of mice in the NFC group, followed by the TFC group showed the highest levels of the anti-inflammatory cytokine IL-10 (*p* < 0.05).

### 3.5 Changes in inflammatory cytokine concentrations in spleen tissue

**Figure 6:**
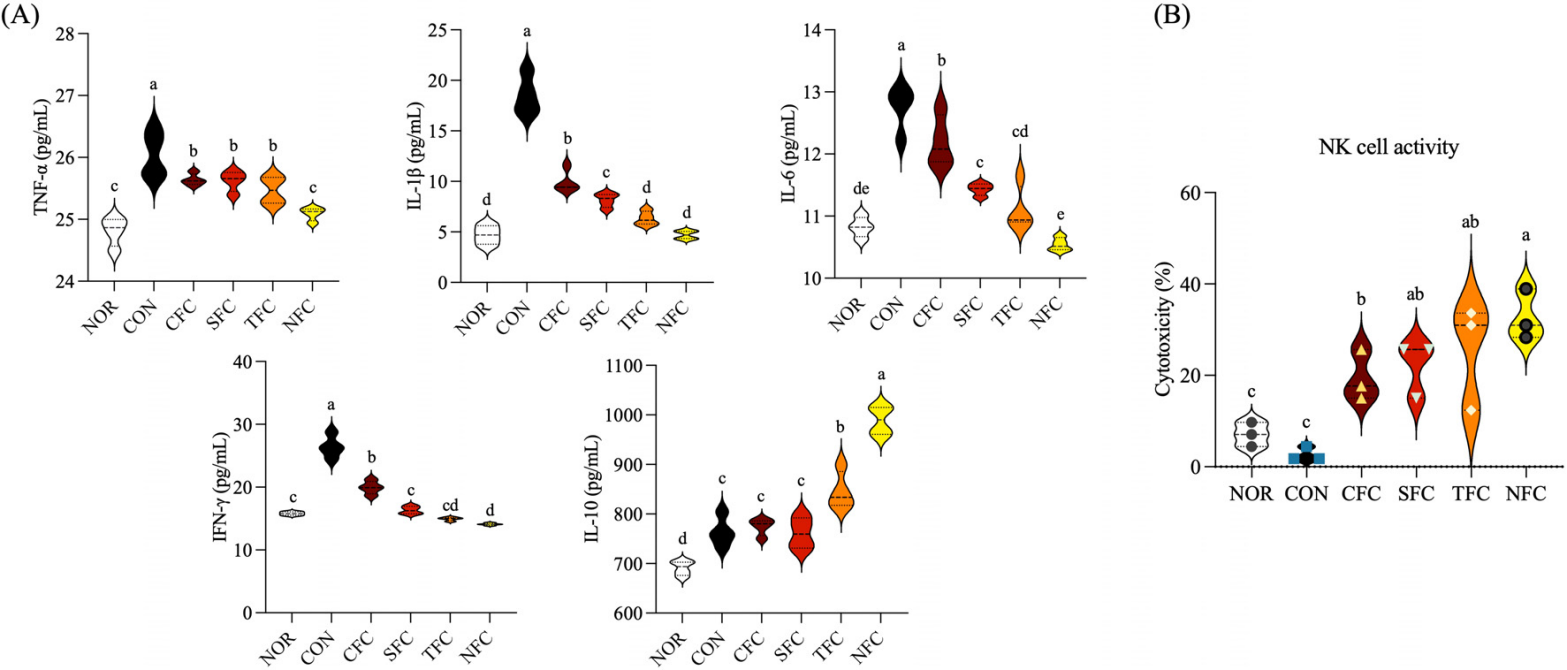
Levels of TNF-α, IL-1β, IL-6, IFN-γ, and IL-10 cytokines **(A)** and NK cell activity **(B)** in the mice splenocytes. NOR: 0.9% saline solution; CON: AOM/DSS + 0.9% saline solution; CFC: AOM/DSS + Conventional Fertilizer Carrot extract 50mg/mL; SFC: AOM/DSS + Seawater Fertilizer Carrot extract 50 mg/mL; TFC: AOM/DSS + Trace element Fertilizer Carrot extract 50 mg/mL; NFC: AOM/DSS + Naturaldream Fertilizer Carrot extract 50mg/mL. Means with the different letters (a-e) above the bars are significantly different by Duncan’s multiple range test (*p* < 0.05).

The levels of inflammatory cytokines in mouse splenocyte culture medium are shown in **Figure 6A**. The CON group had the highest levels of inflammatory cytokines TNF-α, IL-1β, IL-6, and IFN-γ, while feeding mice with NFC resulted in levels similar to those in the NOR group; the other carrot groups exhibited varying degrees of reduction in inflammatory cytokine levels (*p* < 0.05). Meanwhile, the levels of the anti-inflammatory cytokine IL-10 increased in CFC, SFC, TFC, and NFC groups in increasing order.

### 3.6 Natural killer cell activity in mouse splenocytes treated with carrots

To confirm the activity of natural killer (NK) cells in splenocytes, Yac-1 and NK cells were mixed at a 1:5 ratio and cultured. Cell cytotoxicity was evaluated by measuring LDH activity (**Figure 6B**). The activity in the NOR and CON groups was < 10%, while other groups consuming carrot orally exhibited > 20% activity, with the NFC group showing > 30% activity. Additionally, the TFC group showed increased activity, indicating that NK cell activity can be effectively enhanced by crops grown using large amounts of mineral fertilizers.

### 3.7 mRNA expression of inflammation-related genes in mouse liver tissue

**Figure 7:**
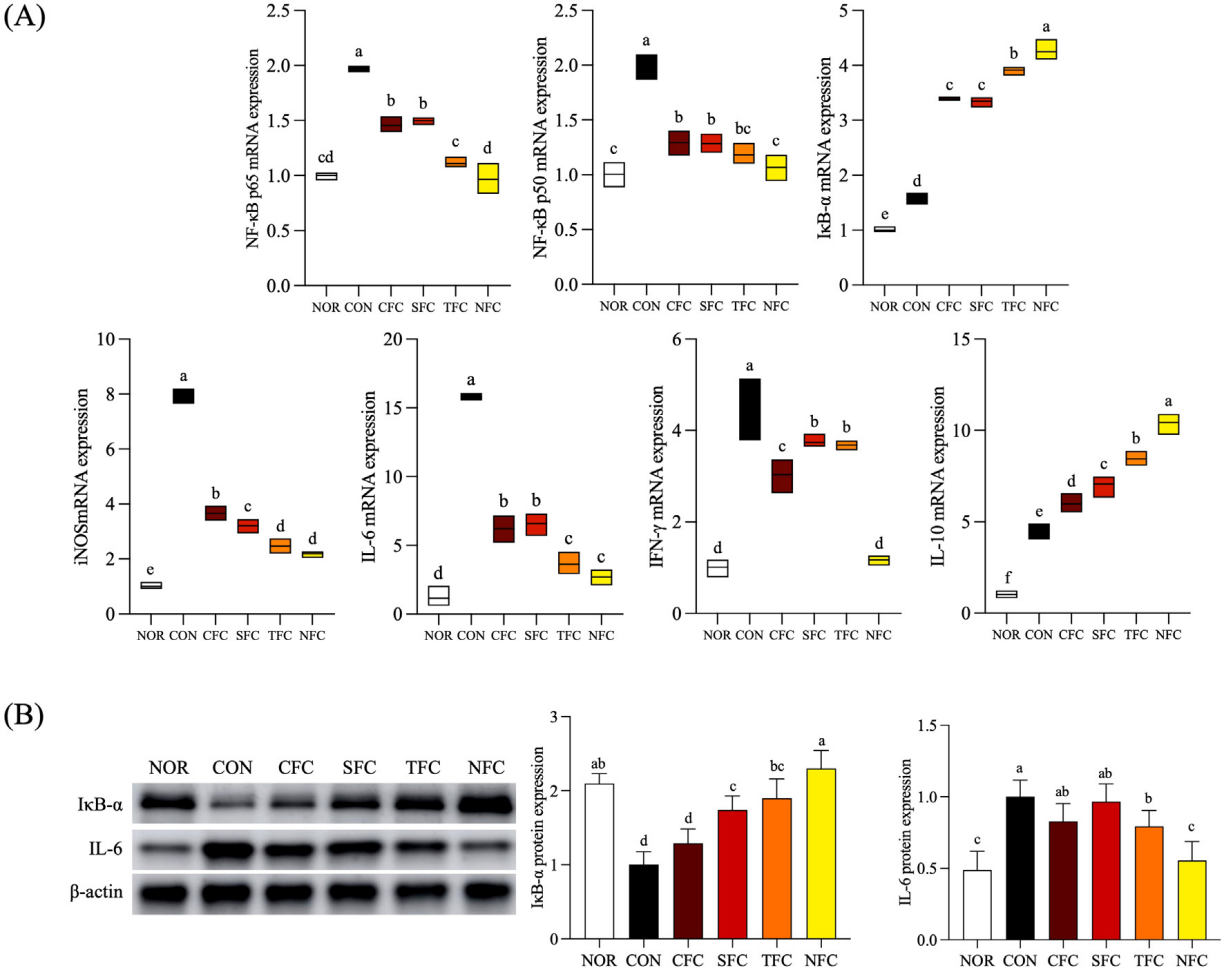
mRNA **(A)** and protein **(B)** expression levels of inflammation-related genes in mice liver tissue. NOR: 0.9% saline solution; CON: AOM/DSS + 0.9% saline solution; CFC: AOM/DSS + Conventional Fertilizer Carrot extract 50mg/mL; SFC: AOM/DSS + Seawater Fertilizer Carrot extract 50 mg/mL; TFC: AOM/DSS + Trace element Fertilizer Carrot extract 50 mg/mL; NFC: AOM/DSS + Naturaldream Fertilizer Carrot extract 50mg/mL. Means with the different letters (a-e) above the bars are significantly different by Duncan’s multiple range test (*p* < 0.05).

**Figure 7A** depicts the mRNA expression levels of inflammation-related genes in mouse liver tissue under carrot treatment. In the CON group, the highest mRNA expression levels were observed for NF-κB p65, NF-κB p50, IL-6, IFN-γ, and iNOS. Compared to the CON group, carrot-treated groups showed a significant reduction in NF-κB p65, NF-κB p50, IL-6, IFN-γ, and iNOS expression, with the lowest expression levels in the NFC group (*p* < 0.05). Additionally, there was an overall increasing trend in IκB-α and IL-10 expression, with the highest levels observed in the NFC group.

### 3.8 Protein expression of inflammation-related genes in mouse liver tissue

The protein expression levels of inflammation-related factors IκB-α, and IL-6 in mouse liver tissue are shown in **Figure 7B**. IκB-α expression was lowest in the CON group, with similar levels observed in the NOR and NFC groups. For IL-6, the expression levels were highest in the CON group compared to the NOR group, with the NFC group approaching NOR level (*p* < 0.05). Furthermore, among these inflammatory genes, mice treated with NFC exhibited expression most similar to the NOR group, followed by the TFC group.

### 3.9 mRNA expression of cell cycle arrest and apoptosis-related genes in mouse colon

**Figure 8:**
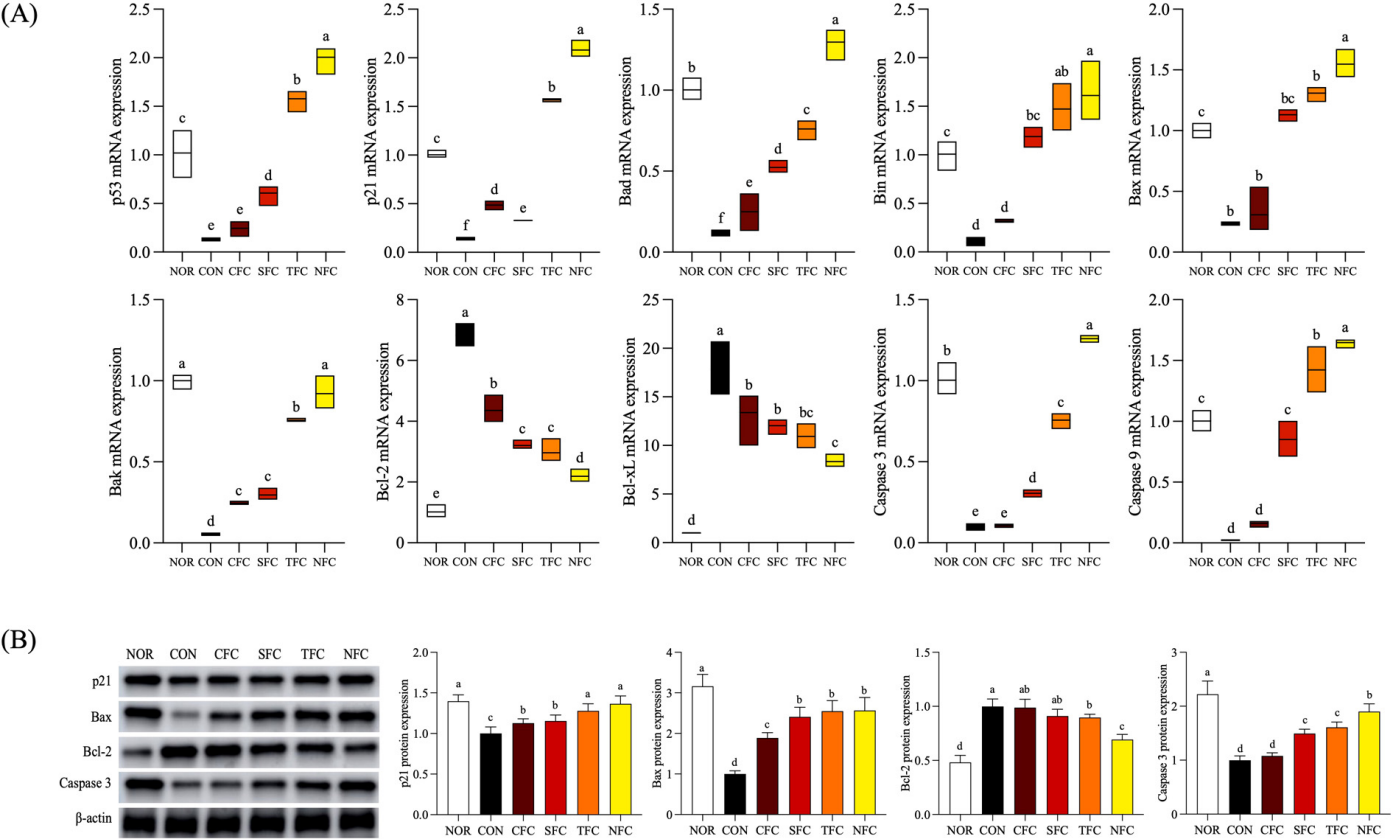
mRNA **(A)** and protein **(B)** expression levels of apoptosis-related genes in mice colon tissue. NOR: 0.9% saline solution; CON: AOM/DSS + 0.9% saline solution; CFC: AOM/DSS + Conventional Fertilizer Carrot extract 50mg/mL; SFC: AOM/DSS + Seawater Fertilizer Carrot extract 50 mg/mL; TFC: AOM/DSS + Trace element Fertilizer Carrot extract 50 mg/mL; NFC: AOM/DSS + Naturaldream Fertilizer Carrot extract 50mg/mL. Means with the different letters (a-f) above the bars are significantly different by Duncan’s multiple range test (*p* < 0.05).

In the mouse colon, the mRNA expression levels of cell cycle arrest-related genes p53 and p21 were lowest in the CON group (*p* < 0.05) and highest in the NFC group (**Figure 8A**). p53 expression levels increased progressively (CFC, SFC, TFC, and NFC), with significant differences between groups (*p* < 0.05). Genes associated with apoptosis promotion, including Bak, Bax, Bad, and Bim, exhibited the lowest expression levels in controls treated with AOM/DSS. Conversely, groups consuming carrots showed increasing expression, with the NFC group showing similar or increased expression than the NOR group. In terms of anti-apoptosis genes Bcl-2 and Bcl-xL, the CON group had the highest expression levels, while the NOR group had the lowest. Furthermore, in carrot-treated groups, expression levels decreased compared to the CON group and showed lowest levels in the NFC group. Additionally, the expression levels of Caspase 9 and Caspase 3, which are involved in the initiation of cell apoptosis, were lowest in the CON group and highest in the NFC group.

### 3.10 Protein expression of cell cycle arrest and apoptosis-related genes in mouse colon tissue

With respect to protein expression of factors related to cell cycle arrest and apoptosis (**Figure 8B**), p21 expression was highest in the NFC group compared to other groups treated with AOM/DSS. Additionally, the expression levels of Bax, and caspase 3, were lowest in the CON group, highest in the NOR group, and closest to the NOR group in the NFC group. Furthermore, compared to the CON group, carrot consumption increased expression, at the highest level in the NFC group. The expression levels of the anti-apoptotic gene Bcl-2 were highest in the CON and CFC groups, and lowest in the NFC group (*p* < 0.05).

### 3.11 Impact of carrots on the intestinal microbiota of CRC mice

**Figure 9:**
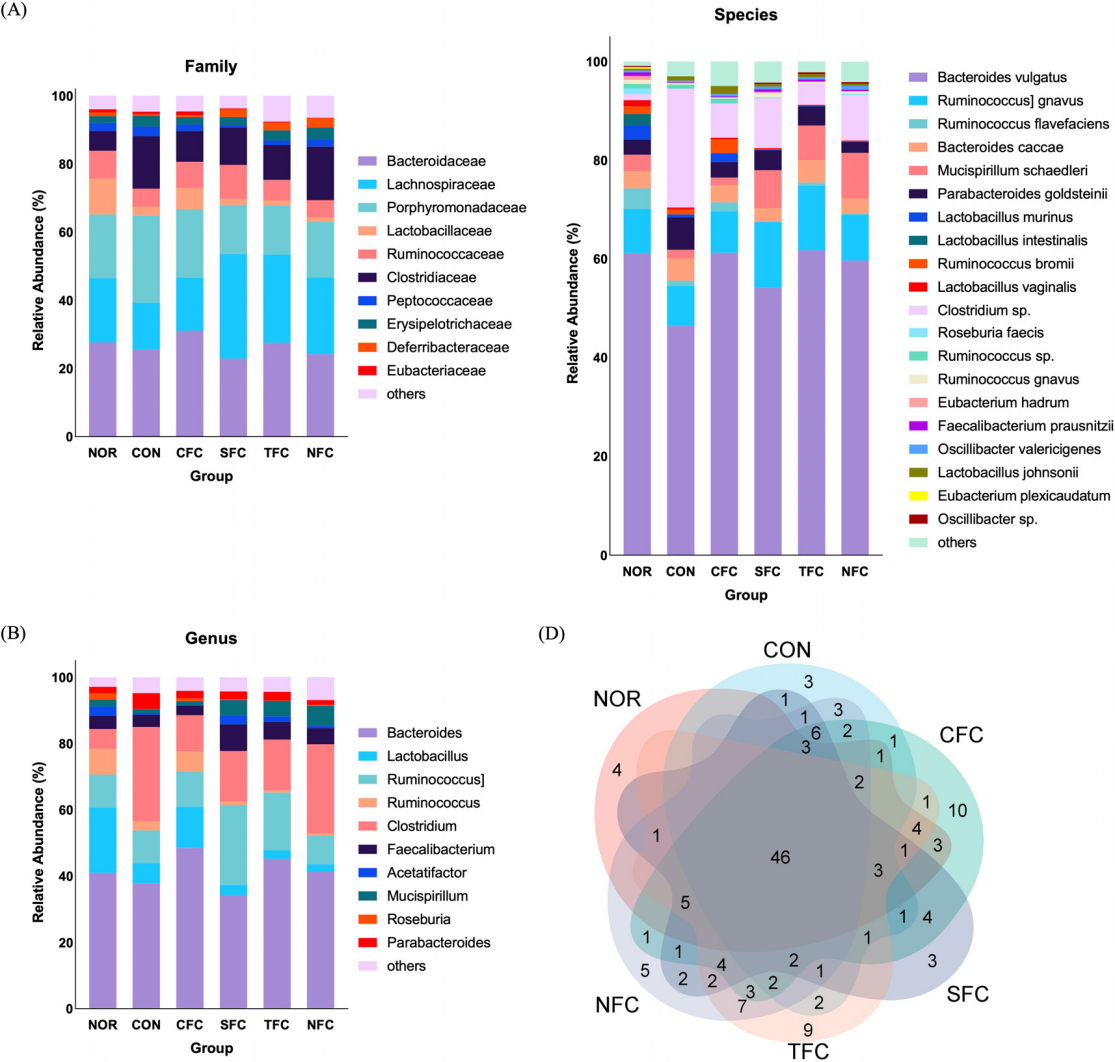
Comparative assessment of Family **(A)**, Genus **(B)**, Species **(C)**, and Venn diagrams **(D)** of mice gut microbiome across different groups. NOR: 0.9% saline solution; CON: AOM/DSS + 0.9% saline solution; CFC: AOM/DSS + Conventional Fertilizer Carrot extract 50mg/mL; SFC: AOM/DSS + Seawater Fertilizer Carrot extract 50 mg/mL; TFC: AOM/DSS + Trace element Fertilizer Carrot extract 50 mg/mL; NFC: AOM/DSS + Naturaldream Fertilizer Carrot extract 50mg/mL.

Finally, we evaluated the impact of carrots on the intestinal microbiota of mice using sequencing. We observed differences in the intestinal microbiota among groups, having both shared and unique operational taxonomic units (OTUs). The normal group had 73 OTUs, the CON group had 81 OTUs, the CFC group had 107 OTUs, the SFC group had 88 OTUs, the TFC group had 96 OTUs, and the NFC group had 99 OTUs, with 46 common OTUs across different samples (**Figure 9D**). Subsequently, comparisons at the family and genus levels (**Figures 9A, B**) revealed the highest relative abundance of *Porphyromonadaceae* in the CON group compared with other groups. In all carrot groups, the relative abundance of *Lachnospiraceae* was higher than in the CON group. At the species level (**Figure 9C**), the relative abundance of *Bacteroides vulgatus* highest in the CON group, followed by Clostridium sp., but with a lower relative abundance of *Mucispirillum schaedleri*.

**Figure 10:**
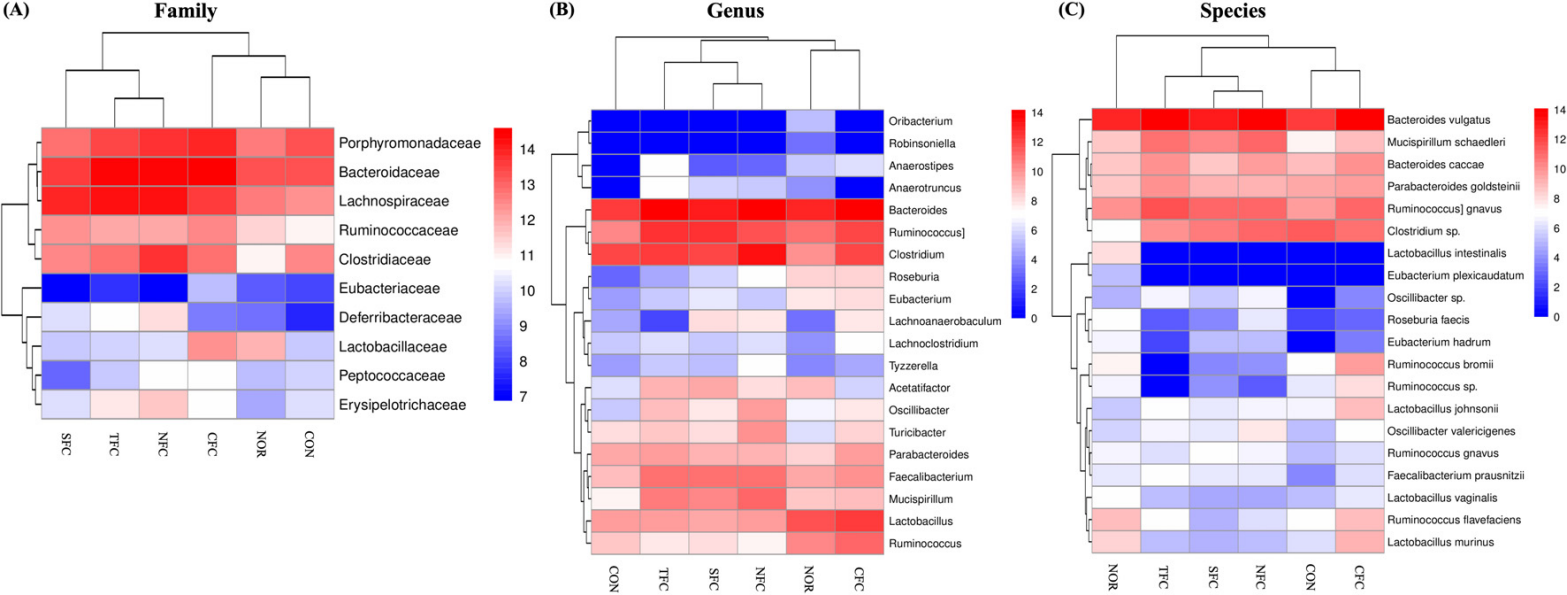
Relative abundance heat map of Family **(A)**, Genus **(B)**, and Species **(C)** showing significant differences and levels. NOR: 0.9% saline solution; CON: AOM/DSS + 0.9% saline solution; CFC: AOM/DSS + Conventional Fertilizer Carrot extract 50mg/mL; SFC: AOM/DSS + Seawater Fertilizer Carrot extract 50 mg/mL; TFC: AOM/DSS + Trace element Fertilizer Carrot extract 50 mg/mL; NFC: AOM/DSS + Naturaldream Fertilizer Carrot extract 50mg/mL.

Furthermore, the relative abundance heatmap (**Figures 10A–C**) showed that at the family level, *Lachnospiraceae*, *Lactobacillaceae*, *Ruminococcaceae*, and *Deferribacteraceae* were the least abundant in the CON group; while *Lachnospiraceae*, *Clostridiaceae*, *Peptococcaceae*, *Erysipelotrichaceae*, *Deferribacteraceae*, and *Sphingobacteriaceae* were the most abundant in the NFC group. At the genus level, Bacteroides, *Faecalibacterium*, *Mucispirillum*, *Roseburia*, and *Oscillibacter* were the least abundant in the CON group; while *Clostridium*, *Mucispirillum*, *Oscillibacter*, *Turicibacter*, and *Tyzzerella* were the most abundant in the NFC group. At the species level, *Bacteroides vulgatus*, [*Ruminococcus*] *gnavus*, *Mucispirillum schaedleri*, *Eubacterium hadrum*, *Faecalibacterium prausnitzii*, *Oscillibacter valericigenes*, and *Oscillibacter* sp. were the least abundant in the CON group; whereas *Mucispirillum schaedleri*, *Oscillibacter valericigenes*, and *Oscillibacter* sp. were the most abundant in the NFC group.

**Figure 11:**
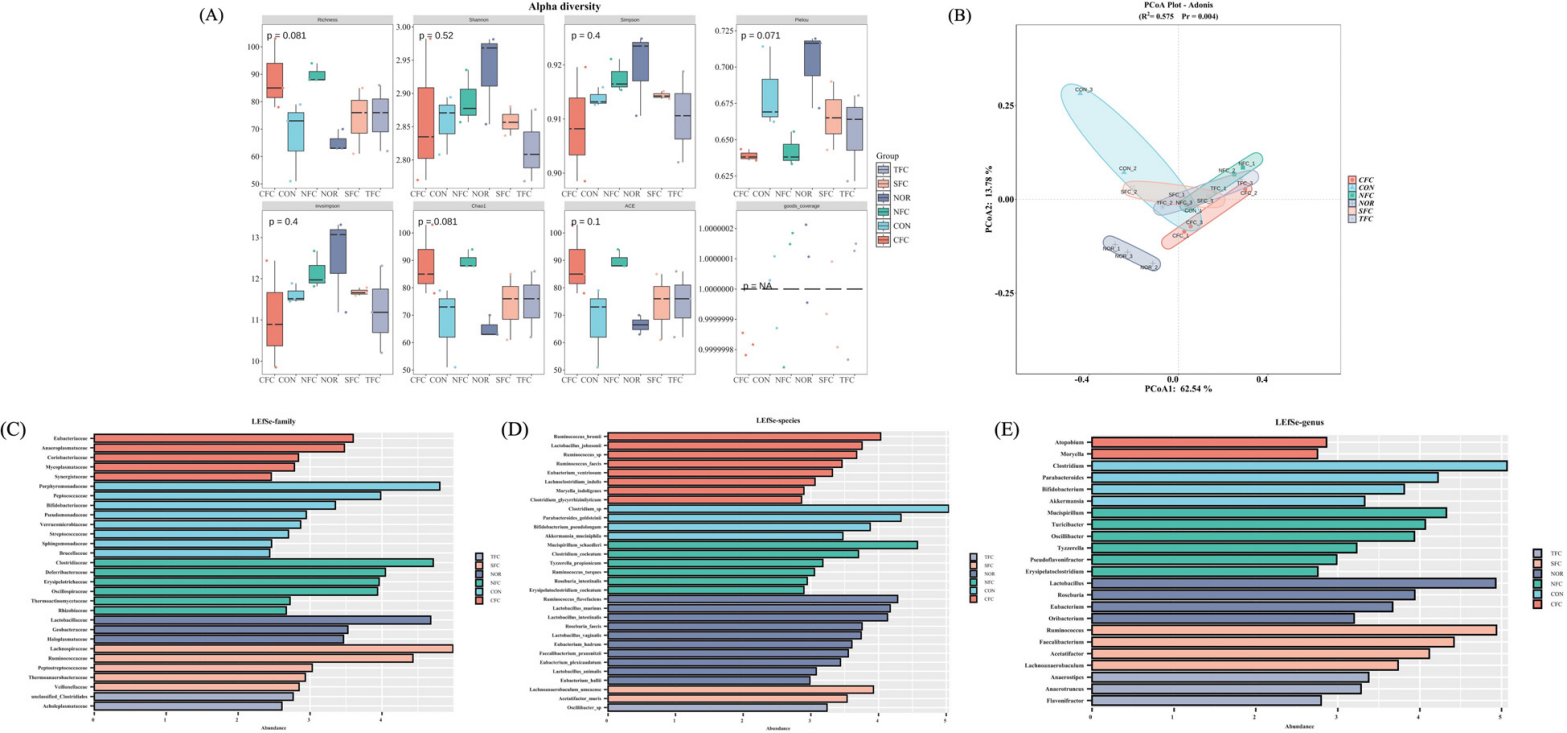
Mice gut microbiome α-diversity analysis **(A)**, β-diversity analysis **(B)**; and LEfSe analysis of differential biomarkers at family **(C)**, genus **(D)**, and species **(E)** levels. NOR: 0.9% saline solution; CON: AOM/DSS + 0.9% saline solution; CFC: AOM/DSS + Conventional Fertilizer Carrot extract 50mg/mL; SFC: AOM/DSS + Seawater Fertilizer Carrot extract 50 mg/mL; TFC: AOM/DSS + Trace element Fertilizer Carrot extract 50 mg/mL; NFC: AOM/DSS + Naturaldream Fertilizer Carrot extract 50mg/mL.

We conducted α-diversity analysis (**Figure 11A**) and β-diversity analysis (**Figure 11B**) to assess species richness and community structure differences. Although most diversity indices (Shannon, *p*=0.52; Simpson, *p*=0.4) showed no significant intergroup differences, the borderline significance of Richness (*p*=0.081) and Chao1 (*p*=0.081) indices suggested potential marginal differences in species richness between groups that did not reach conventional significance thresholds. The Adonis analysis in **Figure 11B** revealed significant microbial community structure divergence between groups (R²=0.575, Pr=0.004, *p*<0.05), with PCoA1 explaining 62.54% of variation and PCoA2 accounting for 13.78%. Non-overlapping distribution ranges of samples from different colored groups (CON, NFC, TFC, etc.) further supported intergroup structural differentiation. Notably, microbial communities in treatment groups (TFC, NFC) showed closer proximity to the NOR group than the CON group in PCoA space, suggesting that differentially cultivated carrots might facilitate restoration of model group microbiota toward normal states.

LEfSe analysis identified differential biomarkers at family, genus, and species levels. At family level (**Figure 11C**), *Porphyromonadaceae* and *Peptococcaceae* were significantly enriched in CON group, while CFC group showed higher abundance of *Eubacteriaceae* and *Anaeroplasmataceae*. Group-specific dominance was observed in NFC (*Clostridiaceae*) and SFC (*Lachnospiraceae*). Genus-level analysis (**Figure 11D**) revealed CON group enrichment of *Clostridium* and *Parabacteroides*, CFC-specific biomarkers (*Atopobium*, *Moryella)*, significant *Lactobacillus* enrichment in TFC, and NFC dominance of *Mucispirillum* and *Turicibacter*. Species-level characterization (**Figure 11E**) demonstrated CON group prevalence of *Clostridium*_sp. and *Parabacteroides*_*goldsteinii*, CFC abundance of R*uminococcus*_*bromii* and *Lactobacillus*_*johnsonii*, TFC-specific *Lactobacillus* species (e.g., *Lactobacillus*_*murinus*), and NFC-specific species including *Clostridium*_*coelatum*. Notably, CON group enrichment of inflammation-associated taxa (*Clostridium* at genus level; *Clostridium*_sp. at species level) contrasted with treatment group proliferation of beneficial bacteria: TFC showed increased *Lactobacillus* and *Bifidobacterium* (genus level), while NFC enriched *Clostridiaceae* (family level). These findings suggest that differentially cultivated carrots may ameliorate AOM/DSS-induced dysbiosis through mechanisms involving the promotion of beneficial bacterial proliferation and intestinal mucosal barrier repair, while suppressing pathogenic bacteria, thereby restoring imbalanced microbial community structures.

## 4 Discussion

In this study, we aimed to investigate the anti-inflammatory and anticancer effects of carrots using various cultivation methods, including deep sea water minerals, on the development of colorectal cancer induced by AOM/DSS. Recently, carrots grown with deep sea water mineral fertilizers (Naturaldream Fertilizer Carrot, NFC) regulate cell cycle inhibition and apoptosis-related pathways better than conventional or organically grown carrots, depending on the cultivation method, thereby inhibiting the growth of HT-29 human colon cancer cells, and improving the growth of C57BL/6 mouse splenocytes. It has been confirmed that it significantly regulates the expression of inflammatory cytokines in cells (26). The AOM/DSS colon carcinogenesis mouse model has been widely used for research on colon carcinogenesis over the past few years (9). Specifically, the AOM/DSS mouse model is commonly employed to replicate the pathogenesis of colorectal cancer (CRC) as observed in patients (35). Therefore, this study utilized the AOM/DSS-induced colon cancer model in C57BL/6 mice. Moreover, these diverse mouse models provide critical frameworks for understanding the pathophysiological mechanisms of not only colorectal cancer but also SARS-CoV-2 infections, sepsis models, and other diseases, thereby aiding in the development of potential therapeutic strategies (36, 37). Oxidative stress is associated with the occurrence and development of cancer (38). Oxidative stress refers to the imbalance of oxidation-reduction within cells, leading to the generation of excessive reactive oxygen species (ROS), including free radicals (such as superoxide anion and hydroxyl radicals) and nonradical oxidants (such as hydrogen peroxide and nitric oxide) (39). When the antioxidant defense system within cells is unable to effectively counteract external or internal oxidative stress, resulting in the generation of ROS exceeding the clearance capacity, it can cause oxidative damage to cellular molecules, including proteins, lipids, and DNA, leading to structural and functional abnormalities in cells, promoting tumor formation, and thus facilitating the development of cancer (40, 41). They possess strong antioxidant capabilities, neutralizing free radicals, regulating cell signaling pathways, influencing gene expression, reducing oxidative stress, and inflammation, thus aiding in the prevention of inflammatory diseases and cancer (42). Therefore, phenol and flavonoid components, which are types of antioxidants, can help reduce oxidative stress and lower the risk of cancer, and carrots, the food used in this study, contain phenolic compounds and flavonoids that effectively regulate intracellular oxidative stress (43). In our study, carrots exhibited high levels of total phenols and total flavonoids, particularly in the NFC variety, showcasing their high antioxidant potential. These results are similar to those shown by Gultekin et al., 2018, where the use of mineral fertilizers significantly affected the production of total phenols, flavonoids, and anthocyanins in grapes (44). In addition, it also showed a similar trend to the changes in phenol and flavonoid content in broccoli by fertilizer identified in previous studies (27).

The AOM/DSS method is a commonly used mouse model for CRC (45). This model simulates the pathological process of human CRC, including epithelial hyperplasia, dysplasia, hyperplastic lesions, and carcinogenesis (46). Mouse body weight can serve as an indicator of metabolism and overall health status, as weight loss may be associated with the occurrence of CRC, which consumes energy from the body, leading to weight loss (47). Similarly in this study, periodic weight loss cycles were observed in the AOM/DSS-induced groups following 2% DSS drinking water treatment. Among the groups that consumed carrots, both the TFC and NFC groups exhibited a tendency to recover body weight, closely aligning with the NOR group. Additionally, colon length is an important indicator of colon health status, as mice with AOM/DSS-induced CRC may experience intestinal inflammation and damage, resulting in shortened colon length (48). Tumor number can reflect the growth and spread of tumors, and in the pathological analysis of colon and spleen tissues, observations of tumor morphology, cell proliferation, and inflammation can provide insights into the pathological changes in tumor tissues (49–51). In this study, NFC treatment helped in the recovery of mouse body weight, significantly inhibiting the shortening of colon length in AOM/DSS-induced CRC mice. Moreover, in the NFC treatment, the colon weight/length ratio was closest to the normal group, and the tumor number the lowest among all induced mouse models. Additionally, the pathological analysis of colon and spleen tissues in mice demonstrated that NFC hindered tumor formation in the colon, and also carrots can enhance the immune function of AOM/DSS-induced CRC mice. These findings suggest that carrots have a certain delaying and inhibiting effect on AOM/DSS-induced CRC, suppressing tumor development and colon shrinkage during CRC progression, and maintaining mouse health, with the most significantly effect in the NFC group.

In the AOM/DSS-induced mouse colorectal cancer model, various inflammation-related factors and signaling pathways play crucial roles (52). In colorectal cancer, high levels of TNF-α may promote tumor growth and spread, activate the NF-κB signaling pathway, enhance cell proliferation, and inhibit apoptosis (53, 54). NF-κB is a key transcription factor involved in inflammation and immune responses and is believed to play an important role in the development of colorectal cancer (55). In particular, NF-κB can induce and regulate the expression of inflammatory cytokine genes (e.g., TNF-α, IL-1β, IL-6, and IL-8) and inflammatory enzymes (e.g., COX-2 and iNOS) (56). This NF-κB is regulated by IκB-α, which is an NF-κB inhibitor that regulates the activation of the NF-κB signaling pathway (57). IL-6 is a pro-inflammatory cytokine that can promote tumor cell proliferation and invasion in colorectal cancer, and it is also associated with the proliferation and self-renewal of tumor stem cells, and IL-1β is primarily a pro-inflammatory cytokine that promotes inflammatory responses, potentially exacerbating inflammation within the tumor microenvironment and facilitating cancer cell growth (58, 59). IFN-γ influences T-cell immune responses and plays a crucial role in tumor immune surveillance and control as an immune-related cytokine (60). iNOS generates nitric oxide and is involved in inflammation and cell death processes as an inducible nitric oxide synthase (61). IL-10 is generally considered an anti-inflammatory cytokine that regulates inflammation and immune responses, offering potential antitumor effects in colorectal cancer (62). In our study, different cultivation methods of carrots show varying effects on the above inflammation-related cytokines and genes. NFC grown with fertilizer mixed with DSWM exhibit significant inhibitory effects on TNF-α, IL-1β, IL-6, IFN-γ, NF-κB, and iNOS in both mouse serum and tissues. Additionally, NFC shows a positive effect on increasing IL-10 and IκB-α levels in mice, indicating that NFC effectively suppresses the deterioration of inflammation in the development of AOM/DSS-induced colorectal cancer, possibly reducing the occurrence of cancer. In addition, regarding the inflammation-inhibiting effect of carrots, it has been confirmed through *in vitro* and *in vivo* studies that chemicals such as falcarinol and falcarindiol can prevent inflammation, and recently, oral ingestion of carrot juice has been shown to reduce the expression of COX-2 and inflammatory cytokines in human blood (63). The superior efficacy of the NFC group may be due to the synergistic effects of DSWM components, such as magnesium and potassium, which may enhance the bioavailability of carrot bioactive compounds like carotenoids and falcarindiol. Furthermore, it has been confirmed that the nutritional quality of carrots improves as soil fertility increases, particularly with the synthesis of falcarindiol being enhanced by phosphate, one of the mineral components (64). Additionally, carrots vary in their ascorbic acid and carotene contents, which are phytochemicals beneficial to human health, depending on agroecological conditions (65). This suggests that fertilizers containing DSWM are more effective in enhancing the phytochemical content of carrots, thereby increasing their anti-inflammatory effects compared to other fertilizers.

NK cells are a crucial subset of lymphocytes with the ability to nonspecifically kill tumor cells, making them early responders in the immune system (66). Additionally, cytokines produced by NK cells, such as interferon and interleukins, impact tumor-related immune responses, affecting tumor growth and metastasis (67). In the AOM/DSS model, the activity of NK cells may influence the functions of other immune cells in the tumor microenvironment, such as T cells and macrophages, thereby affecting the process of immune responses and tumor growth. Carrots are one of the main sources of carotenoids, which play an important role in preventing several diseases and regulating the immune system (68). Particularly, retinoic acid, the biologically active form of vitamin A found in carrots, has been shown to reduce the severity of tuberculosis infection in mice and increase the number of NK cells, T cells, and macrophages in organs such as the lungs and spleen (69). Additionally, the intake of carotenoids like beta-carotene has been confirmed to enhance NK cell activity and overall immune function (70). Likewise, our research suggests that the activity of NK cells tended to significantly increase in the group that consumed carrot extract.

In mouse CRC, various molecules such as p53, p21, Bim, Bad, Bax, Bak, Bcl-2, Bcl-xL, caspase 9, and caspase 3 play crucial roles in the regulation of cell growth, apoptosis, and tumor development. p53 is a crucial tumor suppressor gene often referred to as the “guardian of the genome.” It is activated in response to cellular damage or stress and functions to inhibit tumor development by regulating cell cycle progression, DNA repair, and apoptosis pathways (71). p21 is a direct target gene of p53 and serves as a cell cycle regulator that inhibits cell cycle progression while promoting apoptosis and DNA damage repair (72). In the case of carrots, which contain plant-based active substances such as beta-carotene and polyacetylene, there is a notable induction of cell cycle arrest in leukemia cell lines and a significant increase in p53 expression in HT-29 colon cancer cell lines (73, 74). In this study, the mRNA and protein expression of p53 and p21 in the group that consumed carrots tended to significantly increase compared to the CON group, and the expression was highest in NFC grown with DSWM fertilizer. This demonstrates not only the cell cycle arrest effect of carrots but also suggests that the efficacy of this effect can be enhanced by the type of fertilizer used. Bim, a member of the Bcl-2 protein family, is involved in regulating cell apoptosis. It can induce mitochondrial permeabilization, triggering insulin-like growth factor-1 (IGF-1)-induced growth inhibition and cell apoptosis (75). Bad, another member of the Bcl-2 family, plays a significant role in the apoptosis signaling pathway. Bad binds to the anti-apoptotic proteins Bcl-2 and Bcl-xL, facilitating apoptosis (76). Bax and Bak, also belonging to the Bcl-2 family, are pro-apoptotic proteins that can promote changes in mitochondrial membrane permeability, leading to the release of apoptotic factors and subsequent cell apoptosis (77). Caspases are part of the apoptotic protein family, with Caspase 9 serving as a key protein in initiating the apoptotic signaling cascade and Caspase 3 acting as a crucial protein in the execution of apoptosis by cleaving key intracellular proteins (78). According to several studies, some carrot metabolites have a strong cytotoxic effect only on cancer cells by interfering with key cellular pathways, and can especially regulate various proteins involved in cell proliferation, apoptosis, and inflammation (79). Therefore, it is believed that the group that consumed carrot extract showed a more significant effect on apoptosis-related factors than the CON group. Also, TFC and NFC exerted significant inhibitory effects on cancer development by regulating key factors such as p53, p21, Bim, Bad, Bax, Bak, Bcl-2, Bcl-xL, caspase 9, and caspase 3. These results indicate that TFC and NFC not only increases expression of cell cycle arrest-related genes p53 and p21 but also regulates the expression of apoptosis-related genes, effectively increasing Caspase 9 and Caspase 3 expression, thereby promoting cancer cell apoptosis. Among these, NFC had the strongest inhibitory effects, suggesting that carrots cultivated using the DSWM fertilizers, which promotes NFC production, could serve as a potent source of chemo preventive agents against CRC.

Changes in the intestinal microbiota can affect the immune-inflammatory status of the intestinal mucosa, impact the barrier function of the intestinal mucosa, regulate the expression of inflammatory factors, and influence the growth and apoptosis of intestinal mucosal cells, thereby affecting the development of colon cancer (80, 81). *Porphyromonadaceae* is a common family of intestinal microbiota that is typically present in the intestines of humans and animals. Studies have shown that in a mouse model of colon cancer, an increase in the abundance of the *Porphyromonadaceae* family may be related to changes in the intestinal environment and inflammatory responses, thereby affecting the integrity of the intestinal mucosal barrier and immune response, thus promoting the development of colon cancer (82). As a result of comparing changes in the actual intestinal microorganism community, the community of *Porphyromonadaceae* in the CON group tended to increase compared to the NOR group, and tended to decrease in the carrot consumption groups. Certain species within the *Lachnospiraceae* family contribute to cellulose breakdown and the production of beneficial short-chain fatty acids (83). There is little information on the effects of carotenoid intake on *Lachnospiraceae*, but it has been shown that fucoxanthin, a marine carotenoid, tends to increase in AOM/DSS-induced mouse models (84). Likewise, the *Lachnospiraceae* community in this study tended to decrease in the CON group compared to the NOR group, and showed an increased trend in the TFC and NFC groups to a degree similar to that of the NOR group. Some studies suggest that *Mucispirillum schaedleri* may have a protective effect in the mouse model of colon cancer (85). This microorganism may participate in balancing the intestinal microbial community, promoting the formation and maintenance of the intestinal mucus barrier, and having a positive impact on the development of colon cancer through various mechanisms (86). In particular, *Mucispirillum schaedleri* is believed to help maintain intestinal health, promote the secretion of intestinal mucus, thereby enhancing intestinal defense. In fact, the genus *Mucispirillum* tended to increase in the group that consumed carrots grown with various fertilizers such as SFC, TFC, and NFC compared to the CON group, and its species, *Mucispirillum schaedleri*, showed an increased distribution. This shows that carrots that change depending on the cultivation method can change the distribution of intestinal microorganisms. Some bacteria within the *Clostridium* genus may be beneficial in protecting intestinal mucosal health and suppressing inflammation, thereby playing a positive role in preventing colon cancer formation (87). However, some bacteria of the *Clostridium* genus may increase in colon cancer models and may promote colon tumor formation (88). Therefore, it shows that maintaining it properly can reduce the risk of colon cancer (89). According to our research results, in AOM/DSS-induced colon cancer mice, the relative abundance of *Porphyromonadaceae* and *Clostridium* sp. is high, which may be potential signature bacteria for colon cancer. After carrot treatment, especially with our NFC (name of treatment), the relative abundance of *Lachnospiraceae*, and *Mucispirillum schaedleri* increased, which may be a favorable factor in alleviating the occurrence and development of colon cancer. Additionally, as can be seen in the Venn diagram, the diversity of changing strains is very important for intestinal health.

In this study, we suggest that carrots, especially carrots grown with DSWM fertilizer, exert significant inhibitory effects on AOM/DSS-induced colon cancer, which were confirmed by changes in cell apoptosis, inflammatory responses, and gut microbiome structure. However, there were several limitations in this study. Due to budget and resource constraints in the study design, we were unable to perform repeated time-course measurements or CRC-specific biomarker analysis. These are areas we aim to address in future studies, where we will aim to supplement these limitations and conduct a more comprehensive and accurate evaluation.

## 5 Conclusion

This study aimed to investigate the inhibitory effects of carrots grown with different fertilizers on AOM/DSS-induced CRC in mice. Our results showed that carrots exerted a significant inhibitory effect on tumor development by regulating cell apoptosis and inflammatory responses, with the most significant effect being observed in Naturaldream fertilizer carrot (NFC) grown using deep sea water mineral fertilizer. In addition, NFC significantly increased the expression levels of apoptosis-related genes and proteins in mouse colon tissue while inhibiting the production of inflammatory factors. Further, the gut microbiome analysis in mice treated with carrots significantly differed from those in the control group; the accumulation of active microbiome was closely related to their anti-tumor effects. Overall, our results indicate the potential of carrots as a food source with anti-CRC properties and show that this can be further enhanced depending on cultivation practices.

## Data Availability

The original contributions presented in the study are publicly available. This data can be found here: http://datadryad.org/stash/share/eN3HXL6BeVGVhLsXIWd7wIoWvFCfCy8KuvC3YFDBl4E.
